# Terpenes Combinations Inhibit Biofilm Formation in *Staphyloccocus aureus* by Interfering with Initial Adhesion

**DOI:** 10.3390/microorganisms10081527

**Published:** 2022-07-28

**Authors:** Claudia Salinas, Gladys Florentín, Fátima Rodríguez, Nelson Alvarenga, Rosa Guillén

**Affiliations:** 1 Department of Microbiology, Instituto de Investigaciones en Ciencias de la Salud, Universidad Nacional de Asunción, San Lorenzo 2169, Paraguay; csalinas@qui.una.py (C.S.); meliflorent92@gmail.com (G.F.); farrodriguez288@gmail.com (F.R.); 2 Department of Phytochemistry, Facultad de Ciencias Químicas, Universidad Nacional de Asunción, San Lorenzo 2169, Paraguay

**Keywords:** *S. aureus*, biofilm, terpenes

## Abstract

The biofilm is a conglomerate of cells surrounded by an extracellular matrix, which contributes to the persistence of infections. The difficulty in removing the biofilm drives the research for new therapeutic options. In this work, the effect of terpenes (−)-*trans*-Caryophyllene, (*S)-cis*-Verbenol, (*S*)-(−)-Limonene, (*R*)-(+)-Limonene, and Linalool was evaluated, individually and in combinations on bacterial growth, by assay with resazurin; the formation of biofilm, by assay with violet crystal; and the expression of associated genes, by real-time PCR, in two clinical isolates of *Staphyloccocus aureus,* ST30-t019 and ST5-t311, responsible for more than 90% of pediatric infections by this pathogen in Paraguay. All combinations of terpenes can inhibit biofilm formation in more than 50% without affecting bacterial growth. The most effective combination was (−)-*trans*-Caryophyllene and Linalool at a 500 μg/mL concentration for each, with an inhibition percentage of 88%. This combination decreased the expression levels of the *sdrD,* *spa,* *agr,* and *hld* genes associated with the initial cell adhesion stage and quorum *sensing.* At the same time, it increased the expression levels of the *cap5B* and *cap5C* genes related to the production of capsular polysaccharides. The combinations of compounds tested are promising alternatives to inhibit biofilm formation in *S. aureus.*

## 1. Introduction

*S. aureus* can persist in medical implants, bones, heart valves, and others surfaces through the formation of biofilms, which are cell conglomerates surrounded by an extracellular matrix [1]. These biofilms show an increase of up to 1000 times the antibiotic resistance compared to cells in the planktonic state. The extracellular matrix acts as a physical barrier that decreases antimicrobials’ penetration into the biofilm’s inner layers. Additionally, a limitation in the supply of nutrients leads some cells to a steady state in which antibiotics lose effectiveness, in addition to presenting an increase in the transfer efficiency of plasmids that confer antibiotics resistance [2,3].

There are several genes associated with the formation of biofilm in *S. aureus,* such as genes that encode proteins that participate in the process of adhesion to biotic and abiotic surfaces: proteins with serine-aspartate repeats (*Sdr*), protein A (*spa*), fibronectin-binding proteins (*FnBPA* and *FnBPB*), autolysins (*AtlA* and *AtlE*), bone sialoprotein binding protein (*Bbp*), agglutination factors (*ClfA* and *ClfB*), and collagen adhesion protein (*CNA*), among others [4,5].

Another process associated with the biofilm is the quorum sensing system, with the *agr* (accessory gene regulator) system being the best described for *S. aureus*. In the dispersion stage, the increase in cell density or the accumulation of signal molecules in the medium activates the *agr* system. This system controls the dispersion of cells by increasing the activity of proteases or through molecules with surfactant characteristics called phenol-soluble modulins (PSM, phenol-soluble modulin), among which is the δ-hemolysin (codified by the *hld* gene). An overexpression of δ-hemolysin implies an inhibition in biofilm formation [6,7,8]. Cap genes are also involved in forming biofilm by synthesizing capsular polysaccharides, constituents of the extracellular polymeric substance. In addition, they contribute to the pathogen’s resistance to phagocytosis and thus favor its persistence [9].

Given the difficulty in treating these biofilms with traditional antibiotic agents, other chemical agents have been studied to eradicate them. These new strategies include the use of naturally sourced compounds. These have several advantages, including their abundance, ease of obtaining, and low cost compared to other approaches [10,11,12,13,14]. Within these compounds, terpenes, the main components of the essential oils of numerous plants, have already been shown to possess anti-biofilm activity against *S. aureus* and other bacterial species, also presenting other biological properties: anti-inflammatory, anti-ischemic, antioxidant, insecticide, and antimicrobial [15,16,17].

This work aimed to evaluate the effect of monoterpenes and sesquiterpenes and their combinations on the biofilm formed by highly prevalent community-acquired methicillin-resistant *S. aureus* clones (CA-MRSA) in Paraguay and South America.

## 2. Materials and Methods

### 2.1. Tested Strains

For this study were used the reference strain ATCC 25923 and two isolates of *S. aureus* representative of clones ST30-t019 and ST5-t311, which belong to a strains collection of the Microbiology Department at the “Instituto de Investigación en Ciencias de la Salud”. These clones are responsible for 90% of infections in children caused by this pathogen in Paraguay [18]. These isolates were preserved at −80 °C in vials with half a brain-heart infusion (BHI, Britain, Argentina) supplemented with 15% glycerol. Cultures for subsequent trials were performed on Tryptic Soy Agar (TSA, Becton, Dickinson and Company, Franklin Lakes, NJ, USA).

### 2.2. Monoterpenes and Sesquiterpenes

Stock solutions of the compounds (−)-*trans*-caryophyllene, *(S)-cis*-Verbenol, (*S*)-(−)- Limonene, (*R*)-(+)-Limonene (Sigma-Aldrich, USA), and Linalool (Consolidated Chemical & Solvents LLC, USA) were prepared at a concentration of 40 mg/mL using ethanol (J.T Baker, Phillipsburg, NJ, USA) as a solvent and preserved at −20 °C.

### 2.3. Minimum Inhibitory Concentration (MIC)

The MICs of the antibiotic gentamicin and terpenes were determined using the method described by Sarker et al. in 2007 [19], with some modifications. The test was carried out in a 96-well polystyrene plate, placing in the first column 100 μL of Tryptic Soy Broth (TSB, Becton, Dickinson and Company, USA), supplemented with gentamicin at a concentration of 1 mg/mL or with stock solutions of terpene compounds to reach a final concentration of 20 mg/mL in the first well. In the remaining wells, serial half-dilutions were performed. The last two columns were used as growth control (culture medium with bacterial inoculum or culture medium with solvent ethanol at a concentration of 20 μL/mL with bacterial inoculum) and sterility control (culture medium). A bacterial suspension of 5.10^5^ CFUs was added to each well to reach a final volume of 100 μL. The plate was incubated at 37 °C for 24 h. Ten microliters of resazurin (Sigma Aldrich, Saint Louis, MO, USA) were added to each well, the plate was incubated again at 37 °C, and the reading was performed after 4 h. The lower concentration at which the color remains unchanged is considered the MIC value. The trial was carried out in duplicate, and an average of the values obtained was calculated.

### 2.4. Minimum Inhibitory Concentration for Terpene Combinations

Combinations of two terpenes were tested on the isolated ST5-t311 and ATCC 25923 to show possible synergistic interactions and to evaluate a possible synergistic action between them. The compounds were classified into two groups: exclusive hydrocarbon chain compounds ((−)-*trans*-caryophyllene, (*S*)-(−)- Limonene, (*R*)-(+)-Limonene) and oxygenated compounds (linalool and (*S) -cis*-Verbenol). The combinations were established to include one compound from each group. The determination of MIC was carried out as detailed in the previous section to test terpenes individually. The test was carried out in a 96-well polystyrene plate, placing in the first column 100 μL of Tryptic Soy Broth (TSB, Becton, Dickinson and Company, USA), supplemented with gentamicin at a final concentration of 1 mg/mL or with stock solutions of both terpene compounds to reach a final concentration of 20 mg/mL for each compound (Ratio 1:1) in the first well. In the remaining wells, serial half-dilutions were performed.

The Fractional Inhibitory Concentration Index (FIC) described by Bassolé et al. was calculated to evaluate the possible interactions between compounds. The results were interpreted as follows: synergy (FIC < 0.5), additive effect (0.5 ≤ FIC ≤ 1), indifferent effect (1 < FIC ≤ 4), or antagonism (FIC > 4) [20].

### 2.5. Biofilm Formation Inhibition Assay

The biofilm inhibition assay was performed on a 96-well polystyrene plate with violet crystal staining as described by Qin et al. [21], with some modifications. Three technical replicates were made from each of three biological replicates (cultures of the same isolate made on different days). One colony was used to inoculate 2 mL of TSB medium and then was incubated at 37 °C for 24 h. From this, an inoculum of 0.5 McFarland was prepared in a TSB medium supplemented with 0.25% glucose, using the sterile medium as blank. Then, 190 μL of sterile TSB medium, 5 μL of bacterial inoculum, and the stock solutions were added to each well to reach final concentrations of 500, 250, and 100 μg/mL, except for those wells intended for control without treatment in which five μL of the solvent used were placed, instead of the terpenes. Sterility controls were also included. The plate was incubated at 37 °C for 24 h. After this period, the supernatant was discarded, and three washes were carried out with PBS and then a fixation with 200 μL of ethanol (J.T Baker, USA) per well for one hour. After removing the ethanol, the staining was carried out with violet crystal (Anedra, Buenos Aires, Argentina) at 0.1% for 30 min. The excess violet crystal was removed by three washes with distilled water. The elution was performed with 200 μL of an ethanol/acetone mixture (Cicarelli, Santa Fe, Argentina) (70:30) and a 30-minute mixing in an automatic agitator (Nahita Blue, Navarre, Spain). The optical densities were read using the Multiskan Go spectrophotometer (Thermo Fisher Scientific, Waltham, MA, USA) at 570 nm.

### 2.6. Biofilm Formation Inhibition Assay for Terpene Combinations

The ST5-t311 isolated and ATCC 25923 were used for this assay. According to the procedure described in the previous section, concentrations of 500, 250, and 100 μg/mL were tested for each of the compounds included in the combination (1:1 ratio). All determinations were made in triplicate. The formula described by Qin et al. was used to calculate the inhibition percentages [21].

The criterion published by Yuyama was followed to classify the inhibition percentages. It establishes as elevated biofilm inhibition from 70 to 100%, good from 40 to 69%, and moderate from 20 to 39%. Percentages below 20% are considered inactive [22].

### 2.7. Gene Expression Assay

The combination of (−) *trans*-caryophyllene and Linalool at a 500 μg/mL concentration for each was selected to perform the gene expression assay. Additionally, each compound was tested individually. The test was performed on six-well polystyrene plates. Then, 100 μL of the 20 mg/mL stock solution of the (−)-*trans-*caryophyllene and linalool compounds were added to each well to reach a final concentration of 500 μg/mL for each. A control well was included, in which 100 μL of ethanol was added. One colony was used to inoculate 2 mL of TSB medium, subsequently incubated at 37 °C for 24 h. From this culture, the bacterial inoculum was prepared, in a TSB medium supplemented with 0.25% glucose, to reach a concentration of 3.10^8^ CFU/mL and a final volume of 4 mL per well. Sterility controls were also included. The plate was incubated at 37 °C for 4 h. After that period, the contents of each well were transferred to sterile plastic tubes and centrifuged for 5 min at 1500 rpm. RNA extraction was performed using the commercial Quick-RNA Fungal/Bacterial Kit (Zymo Research, Willoughby, NSW, Australia). After extraction, DNA digestion was performed using the RQ1 RNase-Free DNase kit (Promega, Madison, WI, USA) and complementary DNA synthesis using random primers hexamers (Macrogen, Seoul, Korea).

Six genes related to biofilm formation in *S. aureus* were selected to evaluate changes in gene expression: *spa, sdrD, hld, agrA, cap5B,* and *cap5C.* The presence of the genes in the isolates was verified by whole genome sequencing performed with the Illumina MiSeq sequencer. For amplifying the transcripts of the genes, oligonucleotides and reaction conditions described by Qin and collaborators were used, with some modifications [21]. The gyrase gene was used as a normalizing gene. The reaction was carried out in a final volume of 25 μL using the Rotor-Gene SYBR Green PCR kit (Qiagen, USA) as described by the manufacturer. The cycling parameters were as follows: an initial denaturation at 95 °C by 30 s, followed by 40 cycles of 5 s at 95 °C, 30 s at 60 °C, and 30 s at 72 °C. The 2^−ΔΔCT^ method was used to calculate the normalized relative expression.

### 2.8. Statistics

All the assays were performed independently in biological triplicate. Values are expressed in mean ± SD, and the statistical analysis was performed using GraphPad software (v8.0.1, San Diego, CA, USA). One-way ANOVA and Dunnett’s post hoc test were used to compare the control and treated samples, with a *p*-value less than 0.05 being significant.

## 3. Results and Discussion

### 3.1. Minimum Inhibitory Concentration

The MIC of the terpenes varied between 5 mg/mL and greater than 20 mg/mL, and different susceptibility profiles were observed between the tested strains (Table 1). The MIC for the compound (−)-*trans*-caryophyllene against the isolated ST5-t311 and ATCC 25923 could only be established as superior to 20 mg/mL due to this was the maximum concentration at the compounds was totally soluble. The MIC of gentamicin, used as a control, was less than 2 μg/mL for all the tested strains. These results indicate that the compounds studied do not have good antimicrobial activity. This could be positive for biofilm inhibitor compounds because it decreases the probability of developing resistance [23,24].

Combinations of compounds involving the compound (−)-*trans*-caryophyllene, both with Linalool and with (S)-(*cis*)-Verbenol, presented an additive effect in the strain ST5-t311, but only the combination (−)-*trans*-caryophyllene/Linalool has the same effect in the strain ATCC 25923 (Table 2). Several studies have tested interactions between phenolic terpenes with others of the same type or with alcoholic monoterpenes; however, few studies focus on the evaluation of possible synergistic effects of oxygenated terpenes with those exclusive hydrocarbons [20,25,26,27,28]. Although antagonistic effects would be expected due to differences in solubility, there are several mechanisms postulated through which these interactions can occur: sequential inhibition of the same metabolic pathway, inhibition of protective enzymes, or disruption of the membrane by one compound facilitating the entry of the other [25,29,30,31]. The remaining combinations did not affect the MIC values compared to the values obtained with the individual compounds (Table 1).

The reports presented in this work are the first about the effects of these terpene combinations on *S. aureus*. The effects of some of these compounds have been evaluated in combination with others. For example, the addition of Linalool to certain essential oils may increase their antimicrobial potential [32]. Synergistic interaction between limonene and 1,8-cineole against *S. aureus* has also been described [33]. Another combination that has shown a synergistic effect on this pathogen is geraniol/menthol [34]. Additive-type interactions have been reported with the thymol/carvacrol and cinnamaldehyde/eugenol combinations [35,36].

### 3.2. Effect of Terpenes on Biofilm Formation

**Figure 1:**
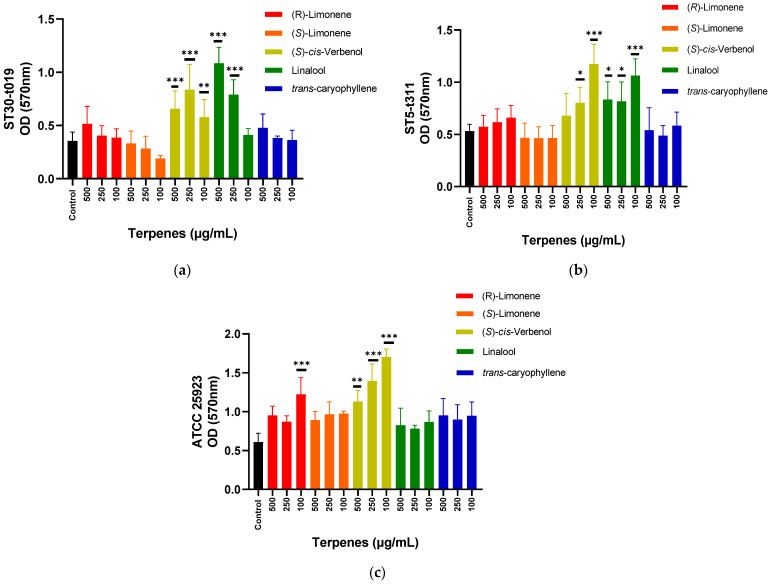
Effect of terpenes on biofilm formation in ST30-t019 isolate (**a**), ST5-t311 isolate (**b**), and ATCC 25923 (**c**). The average of the measurements and the standard deviation are presented in each case. The effect of each concentration is compared with respect to control (one-way ANOVA, Dunnett’s post hoc test). * *p* < 0.05, ** *p* < 0.01, and *** *p* < 0.001 indicate significance.

For the ST30-t019 isolate, an inducing effect on biofilm formation was observed using the compound (S)-*cis*-Verbenol at the three concentrations tested. The linalool compound showed this inducing effect at 500 and 250 μg/mL (Figure 1a). In the case of the ST5-t311 isolate, an inducing effect was observed with (S)-*cis*-Verbenol at 250 and 100 μg/mL and with Linalool at 500, 250, and 100 μg/mL (Figure 1b). For the ATCC strain, an inducing effect was also registered with (S)-*cis*-Verbenol at the three concentrations tested and (*R*)-(+)-Limonene at 100 μg/mL (Figure 1c).

The data obtained in this work for the compound (S)-*cis*-Verbenol constitute the first reports of its effects on biofilm formation in bacteria. In the case of Linalool, various effects of this compound have been observed against the biofilm formed by several bacterial species. Linalool was able to inhibit biofilm formation when it was used at a concentration of 3% (*v*/*v*) against *Shigella flexneri* [37] and reduced the biofilm formation of *Listeria monocytogenes* by more than 50% at a concentration of 0.5% (*v*/*v*) [38]. Linalool can also inhibit the biofilm formation by *Acinetobacter baumannii* and eliminate the biofilm already formed, affecting adhesion capacity and the quorum-sensing system [39]. However, it did not significantly affect the biofilm formed by *Pseudomonas aeruginosa* and enterohemorrhagic *Escherichia coli* (EHEC) [40].

The inducing effect on biofilm formation has also been observed in other studies by using peppermint, sage, and oregano essential oils at concentrations below the MIC against *S. aureus* [41,42]. Essential oils of black pepper and *Mentha suaveolens* recorded antibiofilm activity at a concentration of 1% (*v*/*v*); however, the inhibitory effects are lost by gradually reducing this concentration, and even biofilm formation is induced. Papa et al. suggest, based on these findings, to use essential oils with anti-biofilm activity in conditions in which their concentration is not reduced, such as in the disinfection of surfaces or topical application on the skin or mucous membranes of humans or animals, since their dilution could cause the opposite effect [43].

The inducing activity of these compounds could be exploited in specific models in which biofilm formation is desirable, such as in biological reactors to produce economically important compounds or for the treatment of water or industrial waste. In the latter case, they could be applied to induce the biofilm formation in organisms that do not have this capacity but that have excellent biodegradation properties and, in this way, facilitate the bacterial separation of the treated effluent, always carrying out the necessary tests to determine the optimal concentrations to be used according to the microorganism, given the variable effects observed [44,45].

### 3.3. Effect of Terpene Combinations on Biofilm Formation

All terpene combinations tested in this work inhibited biofilm formation of ST5-t311 isolate by more than 50%. For ATCC 25923, three of the combinations were able to inhibit biofilm formation by more than 50%. In both cases, the most effective combination turned out to be the one involving the compounds linalool and (−)-*trans*-caryophyllene. The most significant effect was observed at the concentration of 500 μg/mL for each compound, with a reduction in biofilm formation of 88% for ST5-t311 and 67% for ATCC 25923, compared to the control condition (Table 3). This combination has already proven effective in inhibiting biofilm formation in *Candida albicans* at a concentration of 0.005% (*v*/*v*) for each compound [46].

These compounds have also been tested in other combinations by other authors. The (−)-*trans*-caryophyllene reduced by less than 30% the formation of biofilm in *L. monocytogenes* and *Salmonella typhimurium* at a concentration of 117 and 133 μg/mL, respectively. However, inhibition percentages exceeded 50% when combined with cinnamaldehyde or eugenol in a 1:1 ratio to their MIC [47]. On the other hand, Linalool acted synergistically with α-longipinene to reduce biofilm formation in *C. albicans* [46]. Linalool’s ability to inhibit the biofilm formed by *S. flexneri* when combined with citral and thymol has also been described. The synergistic effect could be due to one of these compounds can facilitate the entry of the other [37].

Essential oils are a mixture of more than 20 different compounds, so it can be expected that their effects may be due not only to a particular component but to the interaction of several of them. For example, grapefruit essential oil has already been shown to be more effective than its major component (Limonene) in inhibiting the biofilm formation of *P. aureginosa* [48].

The use of Linalool and (−)-*trans*-caryophyllene in cosmetics and as a flavor enhancer has been approved by the Food and Drug Administration (FDA) and the European Food Safety Authority (EFSA) [49,50,51]. Their low toxicity makes them excellent candidates for developing potential products for use as biofilm inhibitors. However, the safety of the combination of these compounds has yet to be evaluated.

### 3.4. Effect of Linalool, (−)-trans-Caryophyllene and Their Combination on the Expression of Genes Associated with Biofilm Formation

#### 3.4.1. Genes Associated with Adhesion

**Figure 2:**
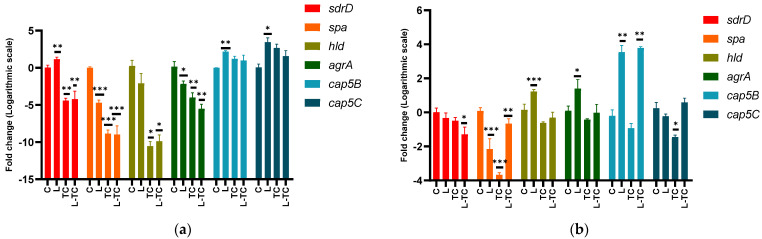
Effect of Linalool, (−)-*trans*-caryophyllene and their combination on gene expression of *sdrD, spa, hld, agrA, cap5B, cap5C* in ST5-t311 isolate (**a**), and ATCC 25923 (**b**). The average of three measurements and the standard deviation are presented in each case. The effect of each concentration is compared with respect to control (one-way ANOVA, Dunnett’s post hoc test) * *p* < 0.05, ** *p* < 0.01, and *** *p* < 0.001 indicate significance. C—control; Lin—Linalool; TC—(−)-*trans*-caryophyllene.

The combination (−)-*trans*-caryophyllene/Linalool reduced the expression of the *sdrD* gene in ST5-t311 isolate and ATCC 25923 (Figure 2). This gene encodes a protein that participates in the initial adhesion stage to surfaces or the extracellular matrix of the host cells and has been shown to mediate the adherence of *S. aureus* to human nasal epithelial cells and other cells by binding to the glycoprotein desmoglein 1. The ability to reduce this gene expression is an important characteristic because its participation in the innate immune system evasion has been demonstrated, thus contributing to increasing the survival of the pathogen in blood and tissues during infections [5,52]

The three conditions tested in both isolates reduced the *spa* gene expression. Protein A, encoded by this gene, is also involved in the evasion of the host immune response by binding to the Fc region of IgG immunoglobulin. Their role in the biofilm has been demonstrated using mutant strains lacking this gene, which significantly reduced their ability to form biofilm. It is presumed to play a role in the initial adhesion; however, its participation mechanism has not been fully elucidated [5,53].

#### 3.4.2. Genes Associated with Biofilm Dispersion

The combination of (−)-*trans*-caryophyllene/Linalool reduced the expression of *agrA* and *hld* in the ST5-t311 isolate but did not affect the expression of these genes in the strain ATCC 25923. Although an increase in the expression of these genes would be expected, at least when using the combination of compounds given the inhibition of the biofilm observed, this result could indicate that the affectation of the *agr* system would not be the mechanism involved in the effect of this combination of compounds. Instead, the reduction in the expression of these genes could indicate a compensatory action, given the decrease in the ability to form biofilm through another mechanism. This decrease in biofilm formation without affecting the quorum sensing agr system could be an extremely desirable quality since its induction could cause the increase in the production of toxins such as hemolysins or Panton-Valentine leukocidin. Another possibility would be that the effect of the combination was because to a blocking of the passage from the early stages of the biofilm to its maturation, thus minimizing its dispersion [7,54,55,56,57,58]

#### 3.4.3. Genes Associated with the Synthesis of Capsular Polysaccharides

The combination of (−)-*trans*-caryophyllene/Linalool increased the expression of *cap5B* but did not affect the expression of *cap5C* in ATCC 25923 and both genes in ST5-t311. The *cap5* locus is involved in the production of capsular polysaccharides, which are components of the cell wall that protect the bacterium against phagocytosis and increase the pathogen’s virulence. However, they can also be recognized by specific antibodies facilitating their elimination. Salimena and collaborators have observed a negative correlation between the production of capsular polysaccharides and the intensity of the biofilm formed [59]. In this work, this correlation was only observed with the gene *cap5B* in the ST5-t311 isolate. It is important to consider the effect of compounds on these genes since a decrease in the expression of capsular polysaccharides is associated with the persistence of infections [60].

Based on the observed gene expression profiles, it could be postulated that the Linalool and (−)-*trans*-caryophyllene combination inhibit the biofilm formation by suppressing the expression of the proteins associated with the adhesion stage, and a compensatory response of the *agr* system could originate from counteracting this inhibitory effect. Given the complexity of biofilm formation, the expression profile of other genes also involved in this process must be investigated to have more refined approximations concerning the mechanism of action.

## 4. Conclusions

Terpene combinations inhibited biofilm formation in the *S. aureus* clones analyzed, with the (−)-*trans*-caryophyllene/Linalool combination being the most effective. The gene expression analysis suggests that this compound combination could affect the biofilm formation adhesion stage. These compounds have the potential to be used to prevent *S. aureus* biofilm-mediated infections, but further investigation would be required.

## Figures and Tables

**Table 1:** Minimum inhibitory concentrations of terpenes.

Terpenes	MIC (mg/mL)		
	ST30-t019	ST5-t311	ATCC 25923
(S)-Limonene	5	15	5
(R)-Limonene	15	20	10
(−)-*trans*-caryophyllene	5	>20	>20
(S)-*cis*-verbenol	20	7,5	5
Linalool	20	7,5	7.5

**Table 2:** Minimum inhibitory concentrations of terpenes combination.

	ST5-t311			ATCC 25923		
Terpenes	MIC (mg/mL)	FIC	Interaction	MIC (mg/mL)	FIC	Interaction
(S)-(−)- Limonene/Linalool	10	2	Indifferent	10	3.3	Indifferent
(S)-(−)- Limonene/(S) -*cis*-Verbenol	7.5	1.5	Indifferent	7.5	3	Indifferent
(R)-(+)-Limonene/Linalool	10	1.8	Indifferent	10	2.3	Indifferent
(R)-(+)-Limonene/(S) -cis-Verbenol	10	1.8	Indifferent	10	3	Indifferent
(−)-*trans*-caryophyllene/Linalool	5	0.9	Additive	5	0.9	Additive
(−)-*trans*-caryophyllene/(S)-*cis*-Verbenol	5	0.9	Additive	5	1.25	Indifferent

**Table 3:** Inhibition percentages of biofilm formed by *S. aureus* ST5-t311 isolated and ATCC 25923 using terpene combinations.

Compounds	Concentration (μg/mL)	ST5-t311		ATCC 25923	
		Percentage of Inhibition	Inhibitory Effect	Percentage of Inhibition	Inhibitory Effect *
(S)-(−)- Limonene /Linalool	500	80.1	Elevated	−40.0 **	-
	250	80.5	Elevated	−21.0 **	-
	100	78.1	Elevated	−8.4 **	-
(S)-(−)- Limonene /(S)-*cis* -Verbenol	500	69.5	Good	−20.7 **	-
	250	63.7	Good	22.1	Moderate
	100	57.1	Good	23.4	Moderate
(R)-(+)-Limonene /Linalool	500	83.8	Elevated	58.0	Good
	250	83.6	Elevated	34.2	Moderate
	100	77.9	Elevated	29.3	Moderate
(R)-(+)-Limonene /(S)-*cis*-Verbenol	500	73.0	Elevated	6.0	Inactive
	250	81.9	Elevated	15.1	Inactive
	100	77.0	Elevated	−21.3 **	-
(−)-*trans*-caryophyllene/Linalool	500	88.1	Elevated	67.2	Good
	250	86.6	Elevated	54.9	Good
	100	74.6	Elevated	9.0	Inactive
(−)-*trans*-caryophyllene/(S)-*cis*-Verbenol	500	85.9	Elevated	64.3	Good
	250	86.7	Elevated	54.3	Good
	100	65.0	Good	19.5	Inactive

* The inhibitory effect was classified as follows: elevated (from 70 to 100%), good (from 40 to 69%), moderate (from 20 to 39%), and inactive (<20%) [22]. ** The negative signs indicate an inductive effect.
